# Tackling antimicrobial resistance in Bangladesh: A scoping review of policy and practice in human, animal and environment sectors

**DOI:** 10.1371/journal.pone.0227947

**Published:** 2020-01-27

**Authors:** Roksana Hoque, Syed Masud Ahmed, Nahitun Naher, Mohammad Aminul Islam, Emily K. Rousham, Bushra Zarin Islam, Shaikh Hassan

**Affiliations:** 1 Centre of Excellence for Health Systems and Universal Health Coverage, BRAC James P Grant School of Public Health, BRAC University, Dhaka, Bangladesh; 2 Laboratory Sciences and Services Division, icddr,b, Dhaka, Bangladesh; 3 Paul G. Allen School for Global Animal Health, Washington State University, Pullman, WA, United States of America; 4 School of Sport, Exercise and Health Sciences, Loughborough University, Loughborough, England, United Kingdom; Johns Hopkins University, UNITED STATES

## Abstract

**Background:**

Antimicrobial resistance (AMR) has become an emerging issue in the developing countries as well as in Bangladesh. AMR is aggravated by irrational use of antimicrobials in a largely unregulated pluralistic health system. This review presents a ‘snap shot’ of the current situation including existing policies and practices to address AMR, and the challenges and barriers associated with their implementation.

**Methods:**

A systematic approach was adopted for identifying, screening, and selecting relevant literature on AMR situation in Bangladesh. We used *Google Scholar*, *Pubmed*, and *Biomed Central* databases for searching peer-reviewed literature in human, animal and environment sectors during January 2010-August 2019, and *Google* for grey materials from the institutional and journal websites. Two members of the study team independently reviewed these documents for inclusion in the analysis. We used a ‘mixed studies review’ method for synthesizing evidences from different studies.

**Result:**

Of the final 47 articles, 35 were primary research, nine laboratory-based research, two review papers and one situation analysis report. Nineteen articles on human health dealt with prescribing and/or use of antimicrobials, five on self-medication, two on non-compliance of dosage, and 10 on the sensitivity and resistance patterns of antibiotics. Four papers focused on the use of antimicrobials in food animals and seven on environmental contamination. Findings reveal widespread availability of antimicrobials without prescription in the country including rise in its irrational use across sectors and consequent contamination of environment and spread of resistance. The development and transmission of AMR is deep-rooted in various supply and demand side factors. Implementation of existing policies and strategies remains a challenge due to poor awareness, inadequate resources and absence of national surveillance.

**Conclusion:**

AMR is a multi-dimensional problem involving different sectors, disciplines and stakeholders requiring a One Health comprehensive approach for containment.

## Introduction

The world is on the verge of sliding back to ‘pre-antibiotic era’ due to evolving resistance against life-saving antimicrobial drugs, with fundamental effect on individual and public health [1]. To combat this emerging global threat in a comprehensive manner, the World Health Organization (WHO) launched a Global Action Plan (GAP) in 2015 based on a ‘One Health’ approach—emphasizing the interdependence of human health, animal health, and the environment [2]. Recently, the interconnectedness of combating antimicrobial resistance (AMR) and achieving Universal Health Coverage (UHC) by 2030 has also been emphasized [3].

The southeast Asia is considered to have the highest risk of AMR among all the WHO regions [4]. Most of the countries in this region have some policy structure (either policy-in-place or policy-in-making) regarding the use of antimicrobials in food animals and fish. For example, the use of antimicrobials is banned as a food additive in some of these countries for rearing food animals, but implementation remains a problem due to the weak regulatory regime [5]. This is also true for policies to prevent contamination of the environment with antimicrobial residues from human and animal use [6].

In many of these countries, antimicrobials are widely available as over-the-counter (OTC) drug, Bangladesh is no exception [7]. The presence of a ‘pluralistic’ health system involving unqualified providers in the informal sector complicates the situation [8]. This is aggravated by the aggressive and unethical marketing practices of the pharmaceutical companies [9]. The regulatory regime in Bangladesh is weak concerning human, technical and logistic capacity to oversee this vast market [10].

Policies and regulations that support appropriate and rational use of antimicrobials are essential for effective interventions to contain the development and spread of AMR. Bangladesh has recently approved a National Action Plan (NAP) for containing AMR, in alignment with the WHO GAP guidelines [11]. For implementation of NAP, detailed information on the current AMR situation in Bangladesh in the human, animal and environment sectors is needed for concerted and coordinated actions across the sectors. Some small-scale studies reported patterns of antimicrobial prescription and use, non-compliance of dosage and development of resistance, and strategies to contain it in different sectors in Bangladesh; however, none so far has looked into the matter comprehensively. This scoping review aimed to fill-in this knowledge gap by examining the nature and extent of the problem in different sectors (human, animal, and environment) in Bangladesh, and exploring how the AMR issue is addressed in policy and practice and associated challenges and opportunities, and how it can be contextualized in the larger paradigm of ‘One Health’ for comprehensive tackling of this public health emergency.

## Methods

To conduct this scoping review, we developed a protocol which specified the study objectives, research questions, inclusion/exclusion criteria, and data sources/search engines to be used (*Google* for unpublished/grey materials; *Google Scholar*, *PubMed*, *and BioMed Central* for peer-reviewed articles) (Table 1). We hand searched for relevant grey materials/unpublished documents from the institutional and journal websites. The key search terms were fixed, for all three sectors such as human, animal and environment (Table 2). A PRISMA extension for Scoping Reviews (PRISMA-ScR) checklist was filled-in to complete this report (S1 Table).

**Table 1:** Literature review protocol.

**Objectives**	Synthesis of evidence on current scenario of antimicrobial use and misuse in all sectors in Bangladesh, level and extent of AMR, programmes/innovations for combating AMR including existing and emerging policies and practices	
	Synthesis of evidence on challenges and opportunities to combat AMR in Bangladesh by identifying barriers and facilitators	
	Make recommendations to inform policy and practice for combating AMR in Bangladesh based upon findings from literature review and from the perspective of One Health	
**Research Questions**	What is the current scenario regarding use of antimicrobials among different population groups that predisposes to the emergence of AMR in Bangladesh? In the food animals and fishes? Contamination of environment and the food cycle?	
	What are the level and pattern of AMR in different geographic regions of Bangladesh?	
	What initiatives/stewardship/surveillance/other programmes have been taken to contain AMR in Bangladesh? The role of One Health approach to address the AMR problem?	
	What are the legal and policy frameworks including national action plan to combat AMR in Bangladesh?	
**Search Strategy**	Inclusion Criteria	AMR related study conducted in Bangladesh (e.g. use and misuse, AM related knowledge and practice including demand and supply side factors, existing AMR policies and strategies, AM use guidelines and stewardship programmes, AMR surveillance, One Health framework, etc.)
		Full text peer-reviewed journal; newspaper articles, blogs and other grey materials e.g., documents in the institutional websites
		Both human, animal and its interconnection with environment-related research
		Language: English
	Exclusion criteria	Excluded countries other than Bangladesh
	Time frame	January 2010 –August 2019
**Data sources**	Peer-reviewed articles	Google Scholar, PubMed, BiomedCentral,
	Grey literature	Google
	Institutional websites	(Ministry of) Health and Family Welfare; Fisheries and Livestock; Agriculture; Environment and Forest; Livestock Research Institute
	Journal websites	Bangladesh Pharmaceuticals Journal, Internal Journal of One Health, Journal of BD Agriculture University, Journal of Dhaka National Medical College Hospital, Bangladesh Journal of Pharmacology, South East Asia Journal of Public Health, Bangladesh Journals Online

**Table 2:** Key terms used for searching electronic databases.

AMR (combined by ‘OR’) (a)	Health sector (combined by ‘OR’) (b)	Geographic location (combined by ‘OR’) (c)
Antimicrobial resistance (AMR) One Health framework, Antibiotic stewardship, AMR surveillance, Knowledge and practices, Current scenario of AMR	Upazila Health Complex, Community Clinics, Primary Healthcare Centers, Tertiary hospitals, Government district hospitals, Private hospitals	Bangladesh

** a,b,c groups were combined with Boolean operator ‘AND’

**Fig 1:**
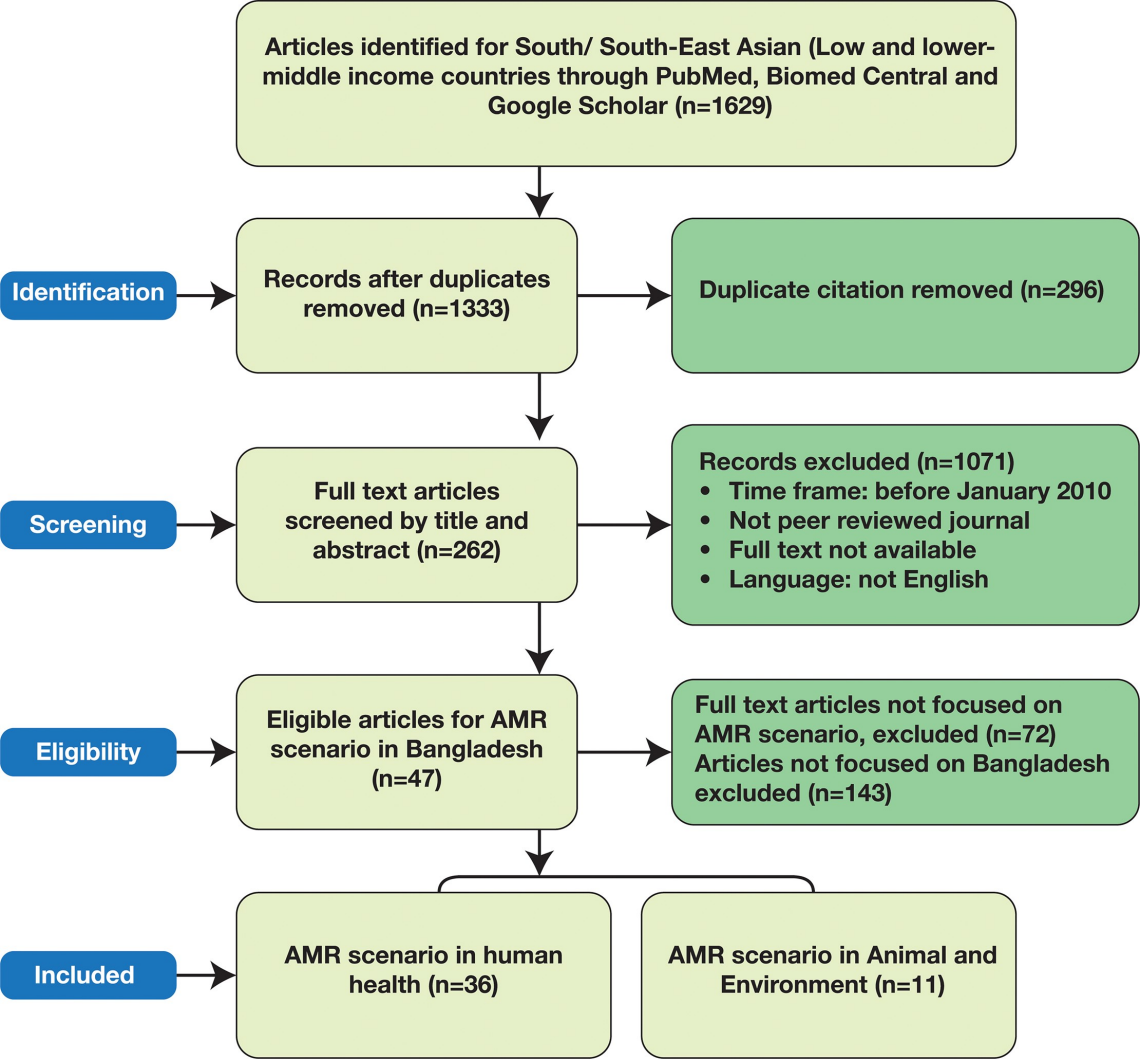
PRISMA diagram for selecting articles on AMR scenario in Bangladesh for inclusion in the analysis.

We followed the Preferred Reporting Items for Systematic Reviews and Meta-Analysis (PRISMA) approach for selection of articles on current AMR situation and relevant policies and practices to be included in the analysis (Fig 1).

### Data extraction and analysis

We used a ‘mixed studies review’ method for synthesizing evidence from qualitative, quantitative and mixed method studies which are useful in understanding complex public health interventions and programmes [12]. A data extraction matrix was used for organizing data, disaggregated into three key themes e.g., current antimicrobial situation (prescribing pattern, use and compliance); perception regarding irrational use of antimicrobials and emergence of AMR (providers, users); and current policies and practices related to antimicrobial use, including relevant sub-themes under each category (Table 3). The study team members critically appraised the selected articles under the guidance of principal investigator, as per PRISMA checklist. Studies that mainly focused on antimicrobial resistance in the human, animal and environment sectors in Bangladesh were included for analysis. Differences in thematic (or sub-thematic) categorization or interpretation of the evidences were resolved by discussion among the study team members under the supervision of the senior investigator to reach a consensus with additional revisions as deemed necessary.

**Table 3:** Framework for analysis (themes and sub-themes).

Theme	Sub-theme
Prescribing patterns and use of antimicrobials including patient compliance	Prescription pattern of antimicrobials and associated factors
	Antimicrobial use in human including compliance
	Antimicrobial use in animal and agriculture
	Antimicrobial contamination of environment
Perception of providers and users regarding rational use of antimicrobials including development of AMR	Perception regarding rational use of antimicrobials among users
	Perception regarding rational use of antimicrobials among providers
	Knowledge and awareness regarding AMR
Current policies and practices to address AMR situation in Bangladesh	Antimicrobial stewardship activities to address antimicrobial resistance in human, animal, and agricultural sectors
	Current policies and strategies to prevent AMR in Bangladesh in humans
	Current policies and strategies to prevent AMR in Bangladesh in livestock, fisheries, agriculture and environment
	National Action Plan (NAP) on prevention of the emergence of, and containing, AMR in Bangladesh

## Results

We used Google, Google Scholar, Pubmed, and BiomedCentral databases for searching relevant literature from Bangladesh. Ultimately, 47 articles (AMR situation in human health = 36 and AMR situation in animal and environment = 11) were included for analysis. Of these, 35 were primary research, nine laboratory-based research, two review papers and one situation analysis report. Again, 19 of the articles on human health dealt with antimicrobial prescribing and/or use, five with self-medication, two on non-compliance of dosage, and 10 were on antibiotics sensitivity and resistance patterns. For AMR scenario in animals and environment, five papers focused on use in animals and six papers on environmental contamination. Besides, current health and drug-related policies and strategies for both humans and animals, and national action plan for containment of AMR in Bangladesh were included through hand search and grey literature search using Google. The findings from the review are described below according to key themes of the study.

### AMR situation in humans: (Table 4)

#### Prescribing pattern, use

Several small-scale studies reported on antimicrobial prescribing and its use, varying by factors such as age and sex. For example, children (66%) were prescribed more antimicrobials than adults (44%) [13], and the rate of prescribing antimicrobials was higher at the extremes of ages [14], and for males [15]. Higher-generationantimicrobials (e.g., ceftriaxone, ciprofloxacin, azithromycin) were prescribed quite frequently [14,16,28,31], especially by the physicians [16]. A study from a slum in Dhaka shows that awareness-building education may improve rational use of antimicrobials, by reducing prescription generation and/or its indiscriminate use [17]. Studies reveal two or more antimicrobials to be commonly prescribed at a time in Bangladesh [14,15,18,19,20,22,30,31], and more antimicrobials to be prescribed in hospital settings compared to community settings [21]. Prescribing antibiotics without laboratory tests was quite common in Bangladesh due to reported lack of testing facilities [18,19,22]. However, sometimes the qualified prescribers (e.g., in-service trainee doctors of medical college hospital) felt confident enough to select an appropriate antimicrobial and prescribe correct dose and duration based on clinical diagnosis [23]. This was also reiterated in a simulated patient study where antimicrobials were prescribed (71%) based on clinical acumen [24]. Qualified prescribers from some secondary and tertiary level hospitals were found to be aware of the treatment guidelines [25], but not those from the upazila (sub-district) hospitals [26]. Sometimes, standard treatment guidelines were not available [27]. Antimicrobials were commonly prescribed for fever, common cold, cough, diarrhea and ARI [16,19,26].

**Table 4:** Summary of studies included for exploring different dimensions of antimicrobial resistance in human health (n = 36).

Author, Year	Type of study	Setting	Themes/sub-themes covered	Key findings
Datta SK et al., 2016 [13]	Cross-sectional (Prescription) study	Prescription pattern was studied from outpatients of a district hospital over three months	Antimicrobial prescribing and/or use	• Children were exposed to antibiotics (66%) more than the adults (44%); cephalosporin was most frequently prescribed (30%) • Physician’s handwriting was legible in only 31% of the prescriptions and didn’t follow standard guidelines
Fahad BM et al., 2010 [14]	Cross-sectional (Treatment) study	Analysis of treatment records of 150 in-patients at a Primary Health Complex in Bangladesh	Antimicrobial prescribing and/or use	• Antibiotic prescription was highest for age groups 5–11 and 65+ years; males had 20% more antibiotics • Two or more antibiotics were prescribed in 8–19% of cases • The highest prescribed antibiotic was Ceftriaxone (30%) followed by Cefixime (19%), and Amoxycillin (17%)
Begum M et al., 2016 [15]	Evaluation study	Evaluation of 300 randomly collected prescriptions from four hospitals in Dhaka	Antimicrobial prescribing and/or use	• 81% prescriptions had two antibiotics • P-lactams were prescribed more frequently (54%) followed by cephalosporin (46%) • 92% of the antibiotics were prescribed by brand names
Sayeed MA et al., 2015 [16]	Cross-sectional (Prescription) study	118 prescriptions were analyzed and 82 local pharmacies surveyed for daily sales of antibiotics in Chittagong city	Antimicrobial prescribing and/or use	• 69% of the prescriptions contained antibiotics; azithromycin and cefixime were most frequently prescribed by the physicians and sold at the pharmacies studied • Antibiotics were prescribed for cough, typhoid, diarrhea, nausea, chronic UTI, RTI, fever and rhinitis
Chowdhury F et al., 2018 [17]	Impact evaluation study	100 randomly selected pharmacies in Dhaka city; post-intervention survey after 6 months following one-day ARI management training	Antimicrobial prescribing and/or use	• For children, dispensing of antibiotics for uncomplicated ARI decreased (30% baseline vs. 21% post-intervention; p = 0.04), and referrals to physicians for complicated ARIs decreased (70% baseline vs. 58% post-intervention; p = 0.03) • For adults, antibiotics dispensing increased among those with complicated ARI (44% baseline vs. 78% post-intervention; p < 0.001)
Haque M (2017) [18]	Multi-country review (including Bangladesh)	The articles were chosen by searching through Google and Google Scholar; Keywords used: antibiotics, use, prescribing, resistance, and the country.	A1ntimicrobial prescribing and/or use	• 50% of outpatient prescriptions in three cities in Bangladesh had antimicrobials; 25% had more than one antibiotic prescribed; 83% were prescribed without any laboratory tests; • Non-compliance was quite high (69%); more than 50% patients stopped antimicrobials as soon as the symptoms started improving • Another study from rural health centers of Dhaka and Chittagong reported that 56% of the physicians prescribe antibiotics in suspected infection, whereas only 33% after confirmatory laboratory tests.
Biswas M. et al., 2015 [19]	Cross-sectional (prescription) study	900 prescriptions from outpatients in three cities of Bangladesh analyzed to elicit practitioners’ prescribing habits and antibiotics-taking behaviour	Antimicrobial prescribing and/or use	• Antibiotics prescribed mainly for common cold and fever, infections, diarrhoea and gonorrhoea; antibiotic was given without any laboratory test in 64% of the cases • Two or more antibiotics prescribed in 25% of prescriptions • The highest prescribed antibiotic was cephalosporin (26%); only 69% of the patients completed the prescribed dosage
Ahmed S. et al., 2018 [20]	Cross-sectional study	A total of 3,570 children aged <5 years presenting with diarrhoea in a tertiary level hospital	Antimicrobial prescribing and/or use	• The rate of antimicrobial prescribing and use at home was 39% compared to 89% during a hospital visit • Children having specific symptoms and signs were more likely to be given antimicrobials in a healthcare setting
Rashid M. et al., 2017 [21]	Cross-sectional study	Children aged <5 years admitted with pneumonia at a private hospital in Dhaka	Antimicrobial prescribing and/or use	• 72.5% of patients received treatment before hospitalization; of them 46% received antibiotics before hospitalization • use of injectable antibiotics was high in the private hospitals which did not follow the WHO standard treatment guidelines
Ata M et al., 2019 [22]	Cross-sectional study	300 prescriptions collected from outpatient departments of a tertiary medical college hospital	antimicrobial prescribing and/or use	• Antibiotics were prescribed in 46% of the cases and injectable antibiotics in 20%; • 80% of the prescriptions had one antibiotic, 20% two antibiotics, and 0.7% three antibiotics. • In 91% of prescriptions, antibiotics given without laboratory test
Hoque R et al., 2015 [23]	Cross-sectional study	Interns of the medicine, gynecology, and surgery departments of a hospital in Chattagram	antimicrobial prescribing and/or use	Majority of the respondents expressed confidence in making an accurate diagnosis, select appropriate antibiotics and advise correct dosage and duration (around 90%) They perceived prescribing inappropriate AM as unethical practice being a doctor (around 60%).
Islam MS et al., 2017 [24]	Simulated patients study	Prescriptions for simulated patients collected (n = 320) from doctors selected randomly from public, private, sub-district health facilities	Antimicrobial prescribing and/or use	• 82% of patients were given more than 2 medicines and 71% were prescribed antibiotics Cost of drugs per prescription was highest at *upazila* (sub-district) level being BDT 301 only and lowest at Dhaka urban area being BDT 265 only. In public hospitals, the cost was BDT 233 only, higher than one would expect.
Arfeen S et al., 2014 [25]	Cross-sectional study; in-depth interviews	Key prescribers of the BSMMU hospital	Antimicrobial prescribing and/or use	• 61% of key prescribers were aware of treatment guidelines; 72% followed institutional guidelines while 22% followed national guidelines • Adherence to guidelines was 29% in case of prescriptions for children and 53% for adults In pediatric patients, adherence was highest for neonatal sepsis (72%) and lowest for bronchial asthma and pneumonia (3%)
Ahmed SM et al., 2012 [26]	Cross-sectional study	30 Upazila Health Complexes (UHC) and 20 urban clinics (UC) in Dhaka	Antimicrobial prescribing and/or use	• An antibiotic was prescribed in 44% of instances Prescribed more frequently for fever (36%) and common cold (26%) than for lower respiratory tract infection, including pneumonia (10%)
Faiz MA et al., 2011 [27]	Review	Articles retrieved through search in Pubmed, Google and Banglajol	Antimicrobial prescribing and/or use	• There is no routine antimicrobial surveillance in Bangladesh • Standard treatment guidelines were not available Antibiotics available as nonprescription drugs in medicine shops
Chodhury A U et al., 2018 [28]	Cross-sectional study	1,100 prescriptions collected through encountering/interviewing outpatients at the exit point and at retail pharmacies	Antimicrobial prescribing and/or use	• 32% did not appear to complete antibiotic courses and 10% took multiple antibiotics • The directions for antibiotic use were incomplete in 40% of cases; dosage form information was incomplete (17%) and absent (5%) • Most frequently prescribed antibiotics was cephalosporin (34%), followed by quinolones (16%), metronidazole (15%), macrolides (13%), and penicillin (7%). Rarely used antibiotics (0.18–2% of prescribed antibiotics) were 1GCs, amoxicillin, co-amoxiclav, gatifloxacin, pefloxacin, and sparfloxacin
Paul TR et al; 2018 [29]	Cross-sectional (prescription) study	329 pediatric prescriptions were analyzed using WHO/ INRUD prescribing indicators	Antimicrobial prescribing and/or use	• 964 drugs were used by the patients with an average 2.93 per prescription. However, none of the drugs was prescribed in generic name. • Only 37% drugs were prescribed from the national essential drug list. Proportions of prescriptions with antibiotics were 83% of which major classes were cephalosporin 45%, β-lactam antibiotics 23%, macrolides 19%, and quinolones 9%.
Shamsuddin AKM et al., 2019 [30]	Cross-sectional study	300 patients (0–15 years) in three major pediatric hospitals of Dhaka, Bangladesh	Antimicrobial prescribing and/or use	• More than two antibiotics were given to a patient in 38.5% of clean cases and 63% of contaminated cases, in combination or sequentially. More than 3 antibiotics were given to a single patient in 47% of contaminated and 80% of dirty categories. More than 80% patients in each group received antibiotic after discharge
Zaman ARBM et al., 2018 [31]	Cross-sectional study	Outpatient department in a Upzilla Health Complex; interview and prescription slips were used for the study.	Antimicrobial prescribing and/or use	• 56% patients were treated with anti-microbials; the majority (77.5%) received two or more antimicrobials in combination The highest prescribed antibiotic was penicillin (42.5%) followed by cephalosporin (35%) and etracycline (32.5%)
Sutradhar K et al., 2014 [32]	Population-based survey	6,000 patients and 580 physicians from 24 Upazila Health Complexes and 112 Union Health Centers in the Dhaka and Rajshahi divisions	Non-compliance /perception regarding use of antimicrobials	• More than 50% patients stop taking the antibiotic as soon as the symptoms disappear • Physicians perceived patient’s noncompliance as the major cause of AMR Patients perceived doctor as incompetent if antimicrobials didn’t work
Saha MR, 2010 [33]	Cross sectional study	350 respondents from four public sector hospitals in Dhaka city	Non-compliance/Knowledge and awareness of antimicrobials use	• 52% stated that they usually did not complete the full course Prevalence of self-medication with antibiotics for cold and fever was high (61%)
Biswas M et al., 2014 [34]	Cross-sectional study	1,300 patients from eight locations of Rajshahi city	Self-medication/Perception regarding antimicrobialsuse	• 27% participants practiced selfmedication with antibiotics; the most frequently used was metronidazole (50%) • Self-medication of antimicrobials related to previous experience (46%) The illnesses for which self-medication was resorted were dysentery, diarrhoea and food poisoning (36%), cold, cough and fever (28%), infection (13%), dental carries and toothache (9%)
Nishat C, 2012 [35]	Cross-sectional study	750 customers were surveyed from six districts of Bangladesh	Self-medication	The self-medication rates for different antimicrobials: ciprofloxacin (3.6%), doxycycline (5%), ampicillin (1%), amoxicillin (2%), azithromycin (1%), cefradine (1*%*) and metronidazole (9%)
Haque et al., 2017 [36]	Cross-sectional study	4,100 patients selected randomly from premises of retail drug shops who came to purchase medicines during January–December 2014	Self-medication	• 23.5% were practicing self-medication with antibiotics; 64.5% were male and 35.5% were female; the most frequent used antibiotic was azithromycin (24%) • About 43% of the study people used antibiotics without prescription; 39% completed scheduled course Reasons for self-medication of antibiotics included advice from traditional healers (41%), prior-experience (33%), knowledgeable of the antibiotics (17.5%) and reduction of doctor’s fees (1.3%)
Rana M et al., 2018 [37]	Cross-sectional study	600 rural adults attending the community clinics in Rajshahi district	Self-medication	Only 17% rural adults had good knowledge about the rational use of antibiotics; those with secondary education and above performed better than their counterparts
Saha et al., 2018 [38]	Cross-sectional study	A section of Geneva Camp population who bought antibiotics in past six months, using a preselected questionnaire	Self Medication/awareness	• 67% bought antibiotics for any disease condition; 89% didn’t buy the full course • Majority didn’t know the name (52%) of the antibiotic bought • 22% used antibiotic for diarrohoea while 14% for fever and cough 60% heard about AMR; 40% from social media
Begum N, 2016 [39]	Antimicrobial sensitivity test	138 Gramnegative uropathogens were isolated	Antibiotics sensitivity and resistance	14% imipenem resistant strains were detected among 138 Gram-negative uropathogens
Mannan A et al., 2014 [40]	Antimicrobial sensitivity test	The antibiotic susceptibility of 70 clinical isolates collected from blood, sputum, urine and pus samples	Antibiotics sensitivity and resistance	• 64% isolates of *Salmonella typhi* were multidrug resistant • Clinical samples were mostly resistant against nalidixic acid for all age groups *Salmonella typhi* was mostly sensitive against gentamycin, chloramphenicol and ciprofloxacin
Rahman MS, 2014 [41]	Antimicrobial sensitivity test	Isolates collected from BSMMU, Dhaka Medcial College, Ibrahim and Rajshahi Medical College	Antibiotics sensitivity and resistance	• 50% of the *Escherichia coli*, *Staphylococcus aureus*, *Pseudomonas* and *Klebsiella* showed resistance against older and common antimicrobials • 70% prescriber mentioned diagnostic uncertainty as causes for increase in antimicrobial prescribing 52% of prescribers opined that physicians prescribe antimicrobial more than the actual need
Ahmed B. et al., 2011 [42]	Antimicrobial sensitivity test	216 culture positive urine samples from Dhaka Medical College Hospital	Antibiotics sensitivity and resistance	• The most common isolate was *E*. *coli* (84%) • Resistance was more in case of amoxicillin (82%) *E*. *coli* was more sensitive to imipenem 94%
Mohammad S, 2010 [43]	Antimicrobial sensitivity test	80 isolates of *E*. *coli* were chosen from public and private hospitals, clinics, diagnostics centers in Dhaka city	Antibiotics sensitivity and resistance	Very low sensitivity of *E*. *coli* towards ampicillin (4%), aztreonam (4%), cloxacillin (5%), nalidixic acid (5%), ciprofloxacin (7.5%), etc. could be attributed to their prevailing usage and abuse in the study area
Tarana N et al., 2018 [44]	Antimicrobial sensitivity test	367 samples, from out and in-door patients of a public sector hospital; blood culture for *Salmonella typhi*	Antibiotics sensitivity and resistance	Prevalence of *Salmonella typhi* was 8%; 70% strains were sensitive to amikacin, 73% to azithromycin, 63% to ceftazidime, 67% to ceftriaxone, and 86.66% to ciprofloxacin; 70% were nalidixic acid resistant *Salmonella typhi*.
Nahar A et al., 2017 [45]	Antimicrobial sensitivity test	A total of 2,541 urine samples collected from suspected urinary tract infection cases and culture and sencitivity was done.	Antibiotics sensitivity and resistance	• Most predominant organism was *Escherichia coli* (86%) followed by Pseudomonas, Enterococci, and Klebsiella (each 4%). *Escherichia coli* showed very high resistance to amoxycillin 95%, cefradin 90%, nalidixic acid, and Klebsiella to amoxycillin 91%, nitrofurantoin 91%.
Ahmed et al., 2018 [46]	Antimicrobial sensitivity test	Laboratory samples from 488 hospitalized patients cultured for *S*. *typhi* and sensitivity test done	Antibiotics sensitivity and resistance	• More than 80% of MRSA isolates were resistant to ampicillin (98%) followed by amoxicillin (94%), cefixime (93.5%) and azithromycin (85%) Maximum sensitivity to tobramycin (86%) followed by gentamicin (85%) and meropenem (79%)
Hasan MJ, 2019 [47]	Antimicrobial sensitivity test	Antibiotic susceptibility data of patients from an elite private hospital and antibiogram data for years 2016, 2017, and 2018	Antibiotics sensitivity and resistance	• The resistance pattern of meropenem, amikacin, ceftazidime and cefepime fluctuated for *E*. *coli*, *Klebsiella pneumonia*, *Acinetobacter baumannii and Pseudomonas aeruginosa* from year-to-year • Polymyxin B and colistin was found to display relatively higher level of sensitivity to these GNB Ceftazidime was always found to have a negative sensitivity trend to *Acinetobacter baumannii* and *Pseudomonas aeruginosa*; on the other hand, amikacin and cefepime were found to display higher level of sensitivity to *P*. *aeruginosa* (6.0 and 7.0, respectively).
Global Antibiotic Resistance Partnership- Bangladesh (GARP): Bangladesh National Working Group [48]	Situation analysis	This review report dealt with up-to-date situation analysis of AMR from a ‘One Health’ perspective	Antibiotics sensitivity and resistance	• In Bangladesh, AMR is precipitated by lack of quality in-patient care as well as brief consultation time, under-value of proper disease diagnosis and use of inappropriate antibiotics • Different evaluation show that almost all antibiotics show resistance to wide range of infectious agents • Low quality drug is responsible for development of AMR as well as causing therapeutic failure and worsening patient condition • In livestock, antibiotics are used for therapeutic purpose and for growth promotion too In spite of widespread use, reliable data on animals are not available in Bangladesh

#### Non-compliance, self-treatment

Patients’ non-compliance with scheduled dosage, and consequently facilitating the emergence of AMR, was a common phenomenon found in the literature reviewed [18,28,32,33]. This may be as high as 50% as patients used to stop taking antimicrobials as soon as the symptoms alleviated [18]. When prescribed antimicrobials did not work in the short-term, patients usually considered the doctors as incompetent [32,33].

The prevalence of self-treatment (taking medicine without consulting a qualified provider) in the reviewed studies was found to be quite high [33–38]. It was found to be common for illnesses such as dysentery, diarrhoea and food poisoning (36%); cold, cough and fever (28%); and presumed infection of some sort (13%) [34]. Reasons behind this self-treatment practice included advice from traditional healers (41%), prior experience with the particular antibiotic for the particular illness (33%), knowledge about antibiotics (17.5%), and waving doctors’ consultation fees (1%). Besides, practice of self-medication was also found to be associated with poverty [38].

#### Antimicrobials sensitivity and resistance

Ten papers reported on antibiotic susceptibility testing and resistance patterns. Resistance against antibiotics like imipenem was found in uropathogens [39]. Susceptibility testing of clinical isolates demonstrated many of the common organisms such as *E*. *coli*, and *S*. *typhi* to be resistant to low-cost and commonly used antibiotics [40–43]. Mannan et al [40] collected 70 types of clinical isolates from samples of blood, sputum, urine and pus and found 64% of isolated *S*. *typhi* to be resistant to multiple antibiotics. Another study conducted in three tertiary level hospitals in Dhaka shows resistance against older and commonly used antimicrobials in *E*. *coli*, *S*. *aureus*, Pseudomonas and Klebsiella infections in 50% of the cases [41]. In 70% of cases, uncertainty about diagnosis was also a reason for prescribing excess antimicrobials. Antimicrobial sensitivity tests conducted on 2,016 culture positive urine samples from a tertiary medical college hospital in Bangladesh found *E*. *coli* in 84% of cases, which was resistant to amoxicillin in 82% of cases [42]. Similar results of low sensitivity of *E*. *coli* against amoxicillin were also observed in another study involving isolates from public and private hospitals, and clinics and diagnostic centres in Dhaka city [43].

In a recent study, Tarana et al [44] reported that *Salmonella typhi* was found sensitive to amikacin (70%), azithromycin (73%), ceftazidime (63%), ceftriaxone (67%) and ciprofloxacin (87%). Another study (2017) found *E*. *coli* in 86% of urine samples collected from a private medical college hospital in Dhaka which was highly resistant to amoxicillin (95%) [45]. More than 80% of MRSA isolates are also found to be sensitive to some macrolides (tobramycin, gentamycin) and carbapenem (meropenem) with resistant to ampicillin, amoxicillin, cefixime and azithromycin [46]. Antibiogram from a private sector tertiary hospital over three years (2016–2018) shows that the last resort antimicrobials (Polymyxin B and Colistin) retained their high sensitivity to Klebsiella, Psudomonos, and *E*. *coli* [47]. A review by GARP- Bangladesh National Working Group provided a descriptive analysis of the AMR situation in different sectors along with recommendations [48].

### Situation in food animals, fisheries, and environment including spread of AMR (Table 5)

A high percentage of poultry meat and eggs meant for human consumption were found to have antimicrobial residues such as tetracycline, ciprofloxacin, enrofloxacin and amoxicillin [49,50]. The prevalence of antibiotic residues varied from >60% in liver, kidney, and eggs to around 50% in breast and thigh samples[49]. In Bangladesh, 3,079 metric tons of poultry manure is produced per day and 50% of this is directly used in aquaculture [51]. For example detectable amount of oxytetracycline residues was traced in 25% *Pangas* fish samples from Sylhet Sadar [52]. This has resulted in the presence of pathogenic bacteria resistant to commonly used antimicrobials in water resources and salad vegetables which are significant from a public health perspective [53–57]. The prevalence of Salmonella species was found to vary between 60% to 78% in different street foods in Chittagong while multidrug resistant Salmonella was found in each of the food items tested [58]. Besides, antimicrobials were found to be used routinely to increase food animal production, resulting in the spread of antibiotic resistant organisms from farms to environment to community [53]. Around 24% of goat farmers mentioned that they were familiar with the term antibiotic but none of them had any idea about antimicrobial resistance nor withdrawal period [58]. The hospitals of Dhaka city are also contributing to the process by discharging untreated medical wastes in the water, resulting in the presence of high-levels of resistant *E*. *coli* in the water [53]. This environmental contamination contributes to wild life infections from humans and animals e.g., the presence of multidrug resistance enterococcus in free-range wildlife and Shiga Toxin- producing *E*.*coli* in Buffalo faeces [59].

**Table 5:** Summary of studies included for exploring different dimensions of antimicrobial resistance in animals and environment (n = 11).

Author(s), Year	Type of study		Setting	Themes/sub-themes	Key findings
Islam S et al., 2016 [49]	Crosssectional study (tissues and eggs of laying hens)		Microbial inhibition test (MIT) and thin layer chromatography (TLC) were used to detect antibacterial residues	Use in animal	• The prevalence of antibiotic residues found 64% in liver, 63% in kidney, 56% in breast muscle, 50% in thigh muscle, and 60% in eggs • Tetracycline (48%), ciprofloxacin (46%), enrofloxacin (40%), and amoxicillin (42%) were found in liver
Sattar et al., 2014 [50]	Cross-sectional study (poultry muscle, kidney, liver)		TLC and ultra-high performance liquid chromatography (UHPLC) method used to detect antibacterial residues	Use in animal	• The residues of tetracycline were 48% in livers, 24% in kidneys, 20% in thigh muscles, and 24% in breast muscles Ciprofloxacin residues were found 44% in liver, 42% in kidneys, 34% in thigh muscles and 30% in breast muscles. Enrofloxacin residues were found 40% in liver, 34% in kidneys, 22% in thigh muscles, and 18% in breast muscle
Hossen M et al., 2015 [51]	Poultry waste management		Trishal *upazila* of Mymensingh district, Bangladesh	Use in animal	• 52% farmer do not use any litter materials • 50% of the farmers sold their litter while some other used as fish feed, soil amendment and for biogas production
Hossain MM et al., 2018 [52]	Cross-sectional study	Oxytetracycline residue in a sample of fishes collected from Sylhet, Bangladesh was detected by High Performance Liquid Chromatography		Use in animal	• Detectable amount of oxytetracycline residues were documented in 25% *Pangas* samples
Islam M et al., 2017 [53]	Comparative analysis of waste water samples from hospital-adjacent areas (HAR)		72 HAR samples tested in Dhaka, Bangladesh	Environmental contamination	• *Klebsiella pneumoniae* (44%) 38 was the predominant bacterial species among blaNDM-1 positive isolates followed by *E*. *coli* 39 (29%), acinetobacter sp., (15%) and enterobacter spp. (9%) • These bacteria were also positive for one or more other antibiotic resistance genes including blaCTX- M-1 (80%), blaCTX-M-15 (63%), 41 blaTEM (76%), blaSHV (33%), blaCMY-2 (16%), blaOXA-48-like (2%), blaOXA-1 (53%) and blaOXA-47- 42 like (60%)
Rashid M et al., 2015 [54]	Epidemiology of antimicrobial resistance ESBL- producing *E*. *coli* in different ecological niches		Water and fresh fecal samples were collected from several locations	Environmental contamination	• 76 *E*. *coli* isolates from water sources were resistant to at least one of the tested antimicrobials • Resistant phenotypes were observed with all antimicrobials except tigecycline, gentamicin, imipenem, and chloramphenicol. Multidrug resistance was observed in 2.6% of Open Bill Stork (OBS) and 37.5% of the water isolates.
Neela FA et al., 2014 [55]	Laboratory-based study	Water sample collected from 4 ponds associated with poultry farms in Puthiya, Bangladesh		Environmental contamination	• Prevalence rate of tetracycline (TC) and ampicillin (AMP) resistant bacteria were 0.24 to 2.59% and 0.16 to 1.0%, respectively in the pond water adjacent to the poultry farm • Higher TC and AMP-resistant bacteria were found in the pond water during rainy season than in the winter
Haque A et al., 2014 [56]	Laboratory-based study	Surface water in Dhaka examined for isolating coliforms and others		Environmental contamination	• *E*. *coli* found most prevalent among isolated Enterobacteriaceae from environmental water • CTX-M-type and SHV-type ESBL genes in isolates that may influence the spread of multidrug resistant pathogenic bacteria
Ahmed T et al., 2014 [57]	Mapped a complete pathogenic profile of the salad vegetables	Dhaka Metropolis, Bangladesh		Environmental contamination	• The pathogenic bacteria present in commonly consumed salad vegetables showed resistance against regular antibiotics
Bhowmik P et al., 2018 [58]	Cross-sectional study	A hospital-based retrospective study using clinical record sheets of goat patients (N = 1405) at a teaching veterinary hospital of Chittagong		Use in animal	• From anthropo-clinical analysis, 24% farmers said that they were familiar with the term ‘antibiotic,’ but no farmer had any ideas about antimicrobial resistance and its withdrawal period Cost of antibiotics was calculated and found to be highest (968.18–1450.04 U.S. Dollars/annum) for Gentasone plus® (gentamicine-sulfadiazine-trimithoprime) and lowest (5.37–8.06 USD/annum) for tylosin.
Hassan M M et al., 2018 [59]	Cross-sectional study	The study was conducted during January-June 2016 in 5 street side markets of Chittagong City Corporation (CCC), Bangladesh		Environmental contamination	• Prevalence of Salmonella spp. varied from 60 to 78% in street food. The study revealed MDR Salmonella (resistance up to 6 of 11 tested antimicrobials) from each of the food items tested. Highest resistances (100%) was detected for ampicillin and amoxicillin and lowest for pefloxacin (around 13%)

### Current policies and strategies to contain AMR in Bangladesh

Quite a number of policies, guidelines, ordinances and laws relevant to containment of AMR in human, food animal, and environment sectors currently exist in Bangladesh (S1 Table). Of these, seven (human = 3; animal and environment = 4) addressed the AMR issue.

#### S1 Table: Table comparing different policies and regulations in the human, animal and environment sectors in Bangladesh

Since the updated National Drug Policy 2016 [60], the government of Bangladesh (GoB) has developed three major policy documents/guidelines with direct implications on the prevention and control of AMR in human sector: i) Pharmacovigilance and Adverse Drug Reaction (ADR) Policy 2017 for monitoring the sale and dispensing of drugs without prescription; ii) Standard Treatment Guideline (STG) for appropriate use of antimicrobials in the sub-districts; and iii) a guideline for antimicrobial stewardship developed by the country’s premiere medical university (S1 Table). These policy documents dealt with different aspects of prevention and control of AMR in clinical settings.

In 2007, the Ministry of Fisheries and Livestock (MoFL) formulated the National Livestock Development Policy where they highlighted inadequate veterinary services and weak implementation of regulatory frameworks as barriers to address AMR in this sector (S1 Table). Specific laws on different aspects of food animals and fish were developed in 2010 where the use of antimicrobials, growth hormones, and pesticides were banned. For violating this law, a person might face up to one year's imprisonment or a fine of up to BDT 50,000 only (USD 650). These documents highlighted issues such as mandatory prescription for antibiotics sale, list of preferred antibiotics for illnesses of a particular system, and 1^st^ and 2^nd^ lines of choice of antibiotics for specific infecting organisms. Banning the general use of antibiotics as a growth factor is emphasized in documents pertaining to the animal sector. To prevent contamination of the environment, specific guidelines are advised for waste management e.g., Guideline for Assessment of Effluent Treatment Plant 2008, Medical Waste Management and Processing Rule 2008, and Environment Protection Act 1995. Interestingly, none of these made any specific reference to the problem of AMR (S1 Table).

Finally, a National Action Plan 2017–2022 for containing AMR in Bangladesh has been prepared by the Disease Control Unit of the Director General of Health Services, MoHFW with a road map for its implementation in alignment with the global plan [61]. The document emphasizes rational use of antibiotics in all sectors through the formulation of standard treatment guidelines, antibiotic stewardship, development of reference laboratories, Good Manufacturing Practice (GMP), Good Pharmacy Practice (GPP), infection prevention and control, and establishment of a comprehensive surveillance.

## Discussion

This scoping review revealed widespread availability of antimicrobials without prescription in Bangladesh, a rising trend in its irrational use in humans and food animals and fisheries, and consequent spread of resistant strains through environmental contamination. The policies and strategies to contain AMR are at an early stage of development in the country and implementation remains a major challenge. This is due to poor awareness about AMR among the policy makers and practitioners, inadequate resources to implement measures to contain AMR, and absence of a comprehensive national surveillance system to monitor the emergence of AMR. The implications of these findings in the context of the emerging ‘One Health’ movement in the country is discussed, including some recommendations for tackling the problem urgently.

### Supply and demand side factors facilitating the emergence of AMR

It is obvious from the findings that the factors underlying irrational and inappropriate use of antimicrobials, and consequent emergence of AMR in Bangladesh, have both supply and demand side perspectives. On the supply side, all categories of the allopathic practitioners whether qualified or not, are prone to use ‘excess’ antimicrobials due to poor awareness regarding its rational use. Other factors contributing to the emergence of AMR include lack of diagnostic facilities, burgeoning private practice and unreasonable demand for antibiotics from patients and ‘caregivers’, especially for children. Besides, there are aggressive marketing strategies of the pharmaceutical companies specifically aimed at generating prescriptions at the cost of patients. This scenario is not unlike that found in other countries of the region such as India [62], Indonesia [63], Malaysia [64], Nepal [65], and Laos [66] irrespective of specialties.

Encouragingly, attempts have been made to overcome the ‘gaps’ in knowledge and use of antimicrobials by these countries which have implication for Bangladesh. For example, the Indian Council of Medical Research (ICMR) brought a qualified group of prescribers in a workshop to enhance their knowledge on AMR and related issues, and involved them in preparing guidelines for rational prescription of antimicrobials [67]. GARP-Nepal is working to outline the components of national strategy for prevention of AMR in Nepal [65]. Indonesia and Malaysia have national level committees to monitor antimicrobial prescription and promote its rational use [68]. These countries hosted a dedicated website for consumer awareness and education regarding rational use of antimicrobials and included schools in the campaign. Besides, the ASEAN countries also included standard treatment guidelines, pharmacovigilence and problem-based pharmacotherapy in their core medical curriculum [68].

In the demand side, rising trend of antimicrobials use resulting from poor consumer awareness on dangers of inappropriate use, use as a stop-gap measure, non-compliance with dosage, and self-medication as observed in Bangladesh are also common in India [69–71], Sri Lanka [72], Nepal [73], Pakistan [74], Indonesia [75,76], and Vietnam [77]. Due to poor level of awareness, sometimes the physicians are pressurized by the patients and/or their caregivers to prescribe antimicrobials especially in urban areas, without any proper indication. In many instances, antibiotics are used as a 'quick & cheap' alternative by both the patients/caregivers and the providers, including use for self-medication [78].

In this review, we found a large proportion of medical practitioners and students in Bangladesh unaware about the guidelines on the use of antimicrobials. The urgency of including this in the graduate curriculum cannot be overemphasized. Coupled with stringent implementation of regulatory measures in the production and distribution of antimicrobials, with appropriate incentives (‘carrot and stick’ approach), will go a long way in mitigating the AMR problem.

### Policies and strategies are there, but implementation remains a challenge

There is no dearth of policies and strategies especially since 2014, but operationalization of these remains a challenge which is true for implementing any public policy in general in Bangladesh [79], including the SDGs [80]. Bangladesh is one of the 100 countries with National Action Plan for containment of AMR developed (as of March 2018), however, a recent review of the Action Plans identified some challenges for its implementation in LMICs like Bangladesh [81]. These include lack of functional mechanism for implementation or coordination, lack of adequate financial and institutional resources for relevant capacity and means for infection prevention and control (e.g., through WASH activities), and building awareness and political commitment. To address these challenges, measures such as mainstreaming of the AMR containment process across sectors, adequate resourcing through national budgets and annual development plans, and inter-sectoral coordination for infection prevention and control are suggested [81].

### The way forward?

The importance of building awareness among the policy-makers for coordinated action across the sectors, updating medical and allied health sciences curriculum for rational use of medicines including antimicrobials, and adequate resourcing with budget and human resources cannot be overemphasized. Again, for the very fundamental task of infection prevention and control in LMICs like Bangladesh, the implementation of WASH activities has an important role to play by reducing the transmission of resistant strains in the environment [82–84]. This is plausible as factors such as poor sewage system; poor infection prevention and control in the hospitals, clinics, education institutions and workplaces; and poor sanitation facilities, and hygiene practices contribute to the spread of resistant strains from environment to food chain. The percentage of resistant isolates may be higher in humans compared to the environment (e.g., animal stool and water sample), but the load (number of resistant isolates/sample) is higher in environment which acts as a reservoir of resistant genes [85]. Though Bangladesh has travelled a long way in controlling open defaecation through much lauded Community Led Total Sanitation (CLTS), the environment remains highly contaminated with faecal matter [86]. In the transition, Bangladesh moved to primitive pit latrines instead of household improved latrine; 60% of the population have onsite sanitation facilities including pit latrines, but onsite sludge management is lacking. In Dhaka, 22% of the households are connected to a sewer system, but only 2% of faecal sludge is estimated to be properly treated. Though majority of the people have access to better quality water for drinking, contamination with microbes, arsenic or salts in pockets is alarming. For example, 80% of piped water is found to be contaminated with *E*. *coli* [86]. The situation is exacerbated by the absence of good hygiene practices which multiplies the effects of unsafe water and sanitation. This importance of WASH interventions is highlighted by a recent Chatam House review of the progress of the AMR situation where the author emphasized the need for more investments in WASH for sustainable containment of AMR over long run in the LMICs [87]. This should alert the policy-makers as provision of sustainable WASH services to the population has implications on overall human development (e.g., stunting, educational attainment) and long-term poverty reduction strategy in Bangladesh.

The problem of AMR is multi-sectoral, multi-disciplinary, and multi-institutional and thus need a coordinated response of the three sectors, adopting a comprehensive approach such as that of One Health. The outbreak of avian influenza in Bangladesh in 2007 exposed the vulnerability of the country to transmission of infection from animals and environment. In 2008, stakeholders in the human, animal and environmental sectors came together to promote the concept of ‘One Health’ in Bangladesh [88]. As a consequence of this movement, the GoB came up with the ‘National One Health Strategy’ in 2012 [89]. In 2016, a One Health Secretariat was established at the IEDCR with staff from three ministries (human health, animal health and environment) and support from the development partners [90]. However, improving awareness among professionals and practitioners across the sectors for consensus actions, and coordination among different sectors and ministries remain a major challenge to move forward. Given the poor state of Bangladesh to ‘prevent-detect-respond’ to health emergencies (i.e., outbreaks of epidemics and pandemics including AMR) as revealed by Global Health Security Index (Bangladesh occupying 113/195 position, scoring 35/100), there is little time to waste [91].

### Limitations

In this scoping review of AMR situation in Bangladesh, we tried to gather maximum available information. However, due to restricted access to some of the databases, the search may not be exhaustive. Besides, there was dearth of published materials on this topic in general, and particularly in some sectors such as the contribution of environmental contamination in initiating and propagating AMR. The included articles were critically appraised but the weighted ratings were not done.

## Conclusion

The factors for the development and transmission of AMR in Bangladesh are deep-rooted in various supply and demand side factors. However, irrational use of antimicrobials in, and beyond, human health sector (e.g., animal health and environment) is aggravating the situation and posing a threat to human health. To address this comprehensively, multi-sectoral and multi-stakeholder efforts are needed based on the strategy of ‘One Health.’ For this, critical awareness of the problem across the sectors and professionals, high level political commitment, intra and inter-ministerial coordination among relevant sectors, and enforcement of regulatory regime are urgently warranted.

## Supporting information

S1 Table Policies and regulations in Bangladesh relevant to prevention and control of AMR. (DOCX)

S2 Table PRISMA-ScR checklist for “Tackling antimicrobial resistance in Bangladesh: A scoping review of policy and practice in human, animal and environment sectors” (PONE-D-19-07275). (DOCX)
