# Supplementary material for: Tackling antimicrobial resistance in Bangladesh: A scoping review of policy and practice in human, animal and environment sectors
Source: PLoS One. 2020 Jan 27;15(1):e0227947. doi: 10.1371/journal.pone.0227947 (PMC6984725; doi:10.1371/journal.pone.0227947)
Supplement: S1 Table — (DOCX) [file pone.0227947.s001.docx]

S1 Table: Policies and regulations in Bangladesh relevant to prevention and control of AMR in the human, animal and environment sectors

| **Regulation/Policy** | **Sector** | **Key features** | **AMR status** |
| --- | --- | --- | --- |
| National Guidelines on the Pharmaco -vigilance System in Bangladesh, 2017  (<http://apps.who.int/me>dicinedocs/documents/s23405en/s23405en.pdf) | Human | - The guideline aims to identify, analyze, and minimize the risks associated with pharmaceutical products. - The guideline gives an overview of what Pharmacovigilance is, how to detect and classify Adverse Drug Reactions. Also describes the reporting system and expected outcome. | AMR issue addressed |
| Development of Clinical Guidelines for Sub-District Level, 2013  *(*[*http://www.rcmc.com*](http://www.rcmc.com)*.bd/wp-content/upl oads/2019/01/Rx-of-top-ten-diseases.pdf)* | Human | - Provides 101 clinical protocols and therapeutic guidelines. - These guidelines provide a more rational basis for referral, promote efficient use of resources, and will also provide a focus for continuing education. | AMR issue not addressed |
| BSMMU Antibiotic guideline, 2012  *(*[*https://image.slidesharecdn.com/antibioticguidelinebvbsmmu-170214101433*](https://image.slidesharecdn.com/antibioticguidelinebybsmmu-170214101433)  /95/antibiotic-guideline-by-bsmmu-1-  638.jpg?cb=1487067350) | Human | - It recommended list of preferred antibiotics for systems (i.e. Central Nervous system, Dentistry, Dermatology, Endocrine system, Gastro Intestinal system, Hepatobilliary system, Infectious disease) and diseases. - It included- likely causative agent, antimicrobial of choice (preferred drug, alternative drug), a committee to upgrade antibiotic guideline of BSMMU | AMR issue addressed |
| The Bangladesh Unani And Avurvedic  Practitioners Ordinance, 1983  (<http://bdlaws.minlaw>.  gov.bd/pdf_part.php?id  =645) | Human | - Establish Bangladesh Board of Unani and Ayurvedic systems of medicine - Maintenance of standards, registration of practitioners, duties and liabilities of registered practitioners, offences, penalty and procedure. | AMR issue not addressed |
| Allopathic System (Prevention of Misuse) Ordinance, 1962 (<http://bdlaws.minlaw>. gov.bd/print sections all.php?id=324) | Human | • No person other than a registered medical practitioner or a person authorized in this behalf by the Government shall prescribe any antibiotic or dangerous drug specified in the rules made under this Ordinance. | AMR issue addressed |

| Animal feed Act, 2010  (<http://www.dls.gov.bd/>site/page/7ebc9c9e-cfaa-4995-b184-9d9928d75710//$fei/M) | Animal | • Animal and fisheries food, quality testing laboratories, Licensing authorities, cancelation regulation, banning low quality animal and fisheries foods, banning use of antibiotic, growth hormone etc. | AMR issue addressed |
| --- | --- | --- | --- |
| Draft National Livestock Development Policy, 2007  (<http://www.dls.gov.bd/>site/page/7ebc9c9e-cfaa-4995-b184-9d9928d75710//$fei/M) | Animal | • National policy for dairy development and meat production; poultry development; veterinary services and animal health; feeds and Fodder | AMR issue not addressed |
| Bangladesh Animal and Animal Product (Livestock), 2005 (<http://www.dls.gov.bd/> site/page/7ebc9c9e- cfaa-4995-b184- 9d9928d75710/ /^IfsilH) | Animal | • Export/import act, customs, quarantine, licensing, fine, compensation related to animal and animal product | AMR issue addressed |
| Veterinary Practitioners Ordinance, 1982  (<http://www.dls.gov.bd/>site/page/7ebc9c9e-cfaa-4995-b184-9d9928d75710//II) | Animal | - Responsibility, publication, registration and privileges of registered veterinary practitioners, degree and diplomas of Veterinary Science. - It is also includes constitutions of council | AMR issue addressed |
| Environment Court Law, 2010  (<https://doe.portal.gov>. bd/site/page/40711011- 9fa2-45f1-bfee- 512c27103284/Environment-Conservation) | Environ  ment | • Establish court to fasten the judging the environment related crimes | AMR issue not addressed |
| Guideline for Assessment of Effluent  Treatment Plant, 2008  (<https://doe.portal.gov>.bd/site/page/42d5ea65-1ca6-4ccd-bc4e-0ee2cd868575/  Environment-Conservation) | Environ  ment | • Environmental Clearance Certificate (ECC) ensures that the industry/project meets all the prescribed standards set by the Bangladesh Government in terms of the quality standards of air, water, noise, odor and other environmental components. | AMR issue not addressed |
| Medical Waste Management & Processing Rules, 2008 (<https://doe.portal.gov>. bd/site/page/42d5ea65- 1ca6-4ccd-bc4e- 0ee2cd868575/Waste- Management) | Environ  ment | - Issuing license for medical waste management, generating awareness through media, communication. - 3 types of license will be given- for package and proper dumping, for collection and transfer, for decompose and refine. - Fine and case for violation of rule | AMR issue addressed |
| Environment Protection Act, 1995 (<https://doe.portal.gov>.bd/site/page/40711011-9fa2-45f1 -bfee-12c27103284/Environ ment-Conservation) | Environment | • Environmental disposal, hazardous  waste, certificate, fees related  clarifications | AMR issue not addressed |
