# Supplementary material for: Tackling antimicrobial resistance in Bangladesh: A scoping review of policy and practice in human, animal and environment sectors
Source: PLoS One. 2020 Jan 27;15(1):e0227947. doi: 10.1371/journal.pone.0227947 (PMC6984725; doi:10.1371/journal.pone.0227947)
Supplement: S2 Table — (DOCX) [file pone.0227947.s002.docx]

**PRISMA-ScR Checklist for “Tackling antimicrobial resistance in Bangladesh: a scoping review of policy and practice in human, animal and environment sectors” (PONE-D-19-07275)**

| **SECTION** | **ITEM** | **PRISMA-ScR CHECKLIST ITEM** | **REPORTED ON PAGE #** |
| --- | --- | --- | --- |
| **TITLE** | | | |
| Title | 1 | Identify the report as a scoping review. | Yes, P-1 |
| **ABSTRACT** | | | |
| Structured summary | 2 | Provide a structured summary that includes (as applicable): background, objectives, eligibility criteria, sources of evidence, charting methods, results, and conclusions that relate to the review questions and objectives. | Yes, P-2, 3 |
| **INTRODUCTION** | | | |
| Rationale | 3 | Describe the rationale for the review in the context of what is already known. Explain why the review questions/objectives lend themselves to a scoping review approach. | Yes, P-5 (First para) |
| Objectives | 4 | Provide an explicit statement of the questions and objectives being addressed with reference to their key elements (e.g., population or participants, concepts, and context) or other relevant key elements used to conceptualize the review questions and/or objectives. | Yes, P-5 (lines 84 -89); also, see Table 1 (Research Qs) |
| **METHODS** | | | |
| Protocol and registration | 5 | Indicate whether a review protocol exists; state if and where it can be accessed (e.g., a Web address); and if available, provide registration information, including the registration number. | Yes, P-6 (archived in the BRAC JPGSPH website; will be made available by the First author on reasonable request) |
| Eligibility criteria | 6 | Specify characteristics of the sources of evidence used as eligibility criteria (e.g., years considered, language, and publication status), and provide a rationale. | Yes, P-7; see Table 1 (search strategy); the literature was searched up-to-date at the time of the study i.e., during July – September 2019) |
| Information sources* | 7 | Describe all information sources in the search (e.g., databases with dates of coverage and contact with authors to identify additional sources), as well as the date the most recent search was executed. | Yes, P-7; see Table 1 (Data Sources) |
| Search | 8 | Present the full electronic search strategy for at least 1 database, including any limits used, such that it could be repeated. | Yes, P-7; see Table 2 |
| Selection of sources of evidence† | 9 | State the process for selecting sources of evidence (i.e., screening and eligibility) included in the scoping review. | Yes, see Fig.1 |
| Data charting process‡ | 10 | Describe the methods of charting data from the included sources of evidence (e.g., calibrated forms or forms that have been tested by the team before their use, and whether data charting was done independently or in duplicate) and any processes for obtaining and confirming data from investigators. | Yes, P-8 (Data extraction and analysis); also, Table 3 (P-8) |
| Data items | 11 | List and define all variables for which data were sought and any assumptions and simplifications made. | Yes, P 7-8 (data extraction and analysis: lines 119-123) |
| Critical appraisal of individual sources of evidence§ | 12 | If done, provide a rationale for conducting a critical appraisal of included sources of evidence; describe the methods used and how this information was used in any data synthesis (if appropriate). | Not done as authentic search engines were used as source |
| Synthesis of results | 13 | Describe the methods of handling and summarizing the data that were charted. | Yes, P-8 (Table 3) |
| **RESULTS** | | | |
| Selection of sources of evidence | 14 | Give numbers of sources of evidence screened, assessed for eligibility, and included in the review, with reasons for exclusions at each stage, ideally using a flow diagram. | See Fig. 1 |
| Characteristics of sources of evidence | 15 | For each source of evidence, present characteristics for which data were charted and provide the citations. | See ref list |
| Critical appraisal within sources of evidence | 16 | If done, present data on critical appraisal of included sources of evidence (see item 12). | Not done. |
| Results of individual sources of evidence | 17 | For each included source of evidence, present the relevant data that were charted that relate to the review questions and objectives. | Yes, P-10-18; 21-24 |
| Synthesis of results | 18 | Summarize and/or present the charting results as they relate to the review questions and objectives. | Yes, P-10-18; 21-24 |
| **DISCUSSION** | | | |
| Summary of evidence | 19 | Summarize the main results (including an overview of concepts, themes, and types of evidence available), link to the review questions and objectives, and consider the relevance to key groups. | Yes, P-27-32 |
| Limitations | 20 | Discuss the limitations of the scoping review process. | Yes, P-32 (Limitations). |
| Conclusions | 21 | Provide a general interpretation of the results with respect to the review questions and objectives, as well as potential implications and/or next steps. | Yes, P-32-33 (Conclusions) |
| **FUNDING** | | | |
| Funding | 22 | Describe sources of funding for the included sources of evidence, as well as sources of funding for the scoping review. Describe the role of the funders of the scoping review. | Yes, It is mentioned in the financial disclosure system. |
